# Clinical impact of endocapillary proliferation according to the Oxford classification among adults with Henoch-Schönlein purpura nephritis: a multicenter retrospective cohort study

**DOI:** 10.1186/s12882-018-1009-z

**Published:** 2018-08-17

**Authors:** Koji Inagaki, Ahmad Baseer Kaihan, Asaka Hachiya, Takaya Ozeki, Masahiko Ando, Sawako Kato, Yoshinari Yasuda, Shoichi Maruyama

**Affiliations:** 1 Department of Nephrology, Nagoya University Graduate School of Medicine, 65 Tsurumai-cho, Showa-ku, Nagoya, 466-8550 Japan; 2 Center for Advanced Medicine and Clinical Research, Nagoya University Hospital, Nagoya, Japan

**Keywords:** Adult, HSPN, Oxford classification, Endocapillary proliferation, JHC

## Abstract

**Background:**

Henoch-Schönlein purpura nephritis (HSPN) is a form of small vessel vasculitis associated with purpura and IgA deposition in the glomeruli. The International Study of Kidney Disease in Children (ISKDC) classification predicts renal prognosis in children with HSPN, but not in adults. Additionally, it is not well known whether the Oxford classification 2016 and/or the Japanese Histologic classification (JHC) are associated with renal outcome. Herein, we investigated the relationship between pathological characteristics and renal outcome among adult patients with HSPN.

**Methods:**

A multicenter retrospective cohort study was conducted in adult patients with HSPN who underwent renal biopsy between 2004 and 2014. Two nephrologists classified each patient according to the Oxford classification 2016, JHC, and the ISKDC classification. Renal outcome was defined by a 30% decline in the eGFR and/or end-stage kidney disease.

**Results:**

We enrolled 74 adult patients with HSPN (mean age, 47.8 ± 17.4 years; mean eGFR, 76.4 ± 25.8 ml/min/1.73 m^2^; median proteinuria, 1.40 [IQR: 0.70–2.38] g/day). During a mean follow-up period of 68.0 ± 33.0 months, fourteen patients (18.9%) reached the renal outcome, and all 14 had received immunosuppressive therapy. The log-rank test revealed that event-free renal survival was significantly shorter in patients with endocapillary proliferation (E1) according to the Oxford classification than in those with E0 (*p* = 0.0072). However, the JHC, ISKDC classification and other Oxford lesions could not demonstrate a significant difference in event-free renal survival. In a multivariate Cox model adjusted for clinical and pathological factors, age (HR, 1.57; 95% CI, 1.12–2.21) and E lesion (HR, 6.71; 95% CI, 1.06–42.7) were independent risk factors for renal outcome.

**Conclusions:**

Endocapillary proliferation is significantly associated with renal outcome in adult patients with HSPN, including those receiving immunosuppressive therapy. Other Oxford classification lesions, JHC, and ISKDC classification were not associated with renal outcome.

**Electronic supplementary material:**

The online version of this article (10.1186/s12882-018-1009-z) contains supplementary material, which is available to authorized users.

## Background

Henoch-Schönlein purpura (HSP) is a form of small vessel vasculitis. According to the European League Against Rheumatism/Pediatric Rheumatology International Trials Organization/Pediatric Rheumatology European Society (EULAR/PRINTO/PRES) classification criteria, the diagnoses of HSP confirmed by the presence of purpura and one of the following manifestations: abdominal pain, arthralgia, renal insufficiency, and leukocytoclastic vasculitis with predominant IgA deposits [1]. About 70–80% of adult patients with HSP develop nephritis (HSPN) manifesting as proteinuria, hematuria, or renal insufficiency [2, 3]. Although, the clinical features of HSPN are different from those of IgA nephropathy (IgAN), the histological and immunofluorescence findings of the two diseases are indistinguishable. Additionally, the glycosylated IgA1 is elevated in both IgAN and HSPN [4]. Thus, it is believed that HSPN and IgAN have common pathogenic mechanisms.

The incidences of IgAN and HSPN are higher among Asian people. In the UK, the annual incidence rates of HSP among black people were lower than those among Asian people [5]. In multinational research, IgAN and HSPN are more common in Asia and Europe, while less common in the USA and Latin America among biopsy-proven kidney diseases [6]. In China, HSPN was the second-most frequently identified disease among biopsy-proven secondary glomerular diseases [7]. Additionally, the length of hospital stay was longer among Asian people than among Caucasians [8]. Thus, HSPN is more common and severe disease among Asians in comparison to other populations.

The renal prognosis of HSPN in adults is worse than that in children [9], and approximately 10% of adults with HSPN reach end-stage kidney disease (ESKD) within 15 years [10]. Clinical findings identified as risk factors for chronic kidney disease (CKD) include old age, impairment of renal function, and massive proteinuria at the time of HSPN diagnosis [3, 9, 10]. According to the International Study of Kidney Disease in Children (ISKDC) classification, the degree of crescent formation predicts renal prognosis [11]. Although, ISKDC classification is associated with renal prognosis in children with HSPN [12], the same association was not observed in adults with HSPN [9, 13].

In 2009, a working group of the International IgAN Network and the Renal Pathology Society developed the Oxford classification to predict the renal prognosis in IgAN patients based on pathological findings [14, 15]. This team identified four histopathologic features associated with renal outcomes independent of clinical parameters: mesangial hypercellularity (M), endocapillary proliferation (E), segmental sclerosis/adhesion (S), and tubular atrophy/interstitial fibrosis (T). Additionally, cellular or fibrocellular crescents (C) were added to the Oxford classification in 2016 [16]. In 2013, the Japanese Histologic classification (JHC) was reported to predict the progression to ESKD in patients with IgAN in Japan [17]. Histologic grades (HGs) in the JHC are based on glomerular lesions which consist of cellular crescent, fibrocellular crescent, fibrous crescent, segmental sclerosis, and global sclerosis. HG I, HG II, HG III and HG IV correspond to < 25%, 25–49%, 50–74%, and ≥ 75% of glomeruli, respectively. JHC was validated for the prediction of renal prognosis in patients with IgAN in 2015 [18, 19].

Only one previous study examined the application of the Oxford classification 2009 in adults with HSPN. Kim et al. reported that Oxford E and T lesions predicted a renal prognosis of ≥30% decline in eGFR or ESKD in 61 adults with HSPN [13]. However there has been no study that validated the HSPN pathology. Additionally, it is unclear whether the Oxford C lesion or JHC is associated with poor renal outcome.

Therefore, we investigated the relationship between pathological characteristics and renal outcome among adult HSPN patients.

## Methods

### Study design and subjects

We performed a retrospective cohort study of adults (≥18 years) with HSPN who underwent a renal biopsy between 2004 and 2014 at fourteen different medical centers (Nagoya University Hospital, Japanese Red Cross Nagoya Daiichi Hospital, Kasugai Municipal Hospital, Anjo Kosei Hospital, Konan Kosei Hospital, Toyota Kosei Hospital, Ichinomiya Municipal Hospital, Handa City Hospital, Toyohashi Municipal Hospital, Yokkaichi Municipal Hospital, Ogaki Municipal Hospital, Chutoen General Medical Center, Gifu Prefectural Tajimi Hospital, and Nagoya Memorial Hospital). We included patients diagnosed with HSPN according to the EULAR/PRINTO/PRES HSP criteria [1] and the presence of IgA-dominant immune deposits on renal biopsy [9]. We excluded patients with less than 12 months of follow-up or with renal biopsy specimens with fewer than eight glomeruli. Patients were followed up until June 2018. During the study period, 89 patients with HSPN underwent a renal biopsy. We excluded ten patients followed up for less than 12 months and five patients with fewer than eight glomeruli in renal biopsy specimens. Seventy-four patients were analyzed.

### Clinical parameters

We analyzed the clinical parameters from the medical records obtained when the patients underwent renal biopsy. A diagnosis of hypertension was defined as blood pressure (BP) values ≥140/90 mmHg or a prescription of antihypertensive drugs. A diagnosis of diabetes was defined as HbA1c levels ≥6.5% or treatment with antidiabetics medications. We calculated the estimated glomerular filtration rate (eGFR) using the modified equation for Japanese [eGFR = 194 × sCr^-1.094^ × age^− 0.287^ × 0.739 (if female)] [20].

### Histological parameters

We stained the renal biopsy samples by periodic acid-Schiff and Masson’s trichrome. Two nephrologists (K.I and AB.K) evaluated the renal biopsy specimens. Each histologic lesion was graded according to the ISKDC Classification, Oxford classification 2016, and JHC [16, 17].

The Oxford classification 2016 is based on the MEST-C score. M0 indicates the involvement of ≤50% of glomeruli with ≥4 cells/mesangial area and M1 indicates the involvement of ≥50% of glomeruli with ≥4 cells/mesangial area. E0 and E1 indicate the absence and presence of endocapillary proliferation, respectively, and S0 and S1 refer to the absence and presence of segmental glomerulosclerosis or tuft adhesion, respectively. T0, T1 and T2 indicate the degree of tubular atrophy or interstitial fibrosis in ≤25%, 26–50%, and > 50% of the cortical area, respectively [14]. C0, C1 and C2 refer to the amount of cellular or fibrocellular crescents of glomeruli as absent, ≤25%, and > 25%, respectively [21].

The ISKDC classification is used to analyze the degree of crescent formation. It includes six histological grades: grade I, minimal glomerular abnormalities without crescents; grade II, mesangial proliferation without crescents; grade III, mesangial proliferation with crescents in < 50% of glomeruli; grade IV, mesangial proliferation with crescents in 50–75% of glomeruli; grade V, mesangial proliferation with crescents in > 75% of glomeruli; and grade VI, membranoproliferative-like lesions [11].

The JHC is based on glomerular lesions in kidney biopsy samples. It includes four HGs, that reflect < 25%, 25–49%, 50–74%, and ≥ 75% of glomeruli. Histologically, the glomerular lesions are seen as follows; cellular crescent, fibrocellular crescent, fibrous crescent, segmental sclerosis, and global sclerosis [17].

### Study outcomes

The primary outcome of this study was a 30% decline in the eGFR from the baseline and/or ESKD.

### Statistical analyses

Parametric and nonparametric variables were expressed as means ± standard deviations (SD) and medians and interquartile ranges (IQRs). They were compared using Student’s t-test and the Mann–Whitney *U* test. Categorical variables were expressed in percentages and were compared using the Fisher’s exact test. The Spearman correlation coefficient was used to determine the relationship between two histologic lesions. Kaplan–Meier survival curves were used to analyze freedom from the primary outcome in patients grouped according to the following classification: M0/1, E0/1, S0/1, T0/1 + 2, C0/1/2, ISKDC (grade I + II/III + IV), and JHC (HG I/II + III + IV). We used the univariate Cox regression to determine the factors predicting the primary outcome. Proteinuria was measured using time-collection urine, but we were not able to measure time-collection urine in four patients. Thus, they were assessed by calculating the spot urine protein-creatinine ratio. Variables with *P* values < 0.30 in the univariate analysis were examined in the multivariate analysis. These results were expressed as hazard ratios (HRs) with 95% confidence intervals (CIs). P values < 0.05 were considered statistically significant. All statistical analyses were performed with EZR (Saitama Medical Center, Jichi Medical University, Saitama, Japan). EZR is a graphical user interface for R statistics (The R Foundation for Statistical Computing, Vienna, Austria) [22].

## Results

### Baseline clinical and pathological characteristics

The baseline clinical characteristics are shown in Table 1. The mean age was 47.8 ± 17.4 years, and 47.3% were male (the detailed data were shown in Additional file 1). The mean eGFR was 76.4 ± 25.8 mL/min/1.73 m^2^, and the median proteinuria was 1.40 [IQR: 0.70–2.38] g/day. The median duration between lower limb purpura and the time of renal biopsy was 53 [IQR: 25–168] days. After a median kidney biopsy procedure period of 12 [IQR: 1–25] days, oral prednisolone (PSL) was prescribed to 61 (82.4%) patients.

**Table 1:** Baseline clinical and pathological characteristics in 74 adults with HSPN

	All (*n* = 74)
Clinical parameters	
Age, years	47.8 ± 17.4
Male/female	35 (47.3) /39 (52.7)
Body mass index, kg/m^2^	23.3 ± 4.41
Diabetes	7 (9.5)
Hypertension	26 (35.1)
Systolic blood pressure, mmHg	125.7 ± 16.9
Diastolic blood pressure, mmHg	74.1 ± 12.2
Gross hematuria	13 (17.6)
Abdominal symptoms	25 (33.8)
Arthralgia	18 (24.3)
eGFR, mL/min/1.73 m^2^	76.4 ± 25.8
Total cholesterol, mg/dL	216.4 ± 62.4
Serum uric acid, mg/dL	5.3 [4.1–7.1]
Serum IgA, mg/dL	333 [258–453]
IgA/C3 ratio	3.01 [2.18–3.76]
Proteinuria, g/day	1.40 [0.70–2.38]
Proteinuria, g/gCr	1.87 [1.09–3.32]
U-RBC ≥30/HPF	38 (51.4)
Days between purpura onset and renal biopsy	53 [25–168]
Pathological parameters	
Oxford M0/M1 lesion	69 (93.2)/5 (6.8)
Oxford E0/E1 lesion	36(48.6)/38 (51.4)
Oxford S0/S1 lesion	36(48.6)/38 (51.4)
Oxford T0/1/2 lesion	56 (75.7)/14 (18.9)/4 (5.4)
Oxford C0/1/2 lesion	22 (29.7)/35 (47.3)/17 (23.0)
JHC I/II/III/IV	39 (52.7)/27 (36.5)/8 (10.8)/0 (0)
ISKDC grade I/II/III/I*V*/V	6 (8.1)/14 (18.9)/52 (70.3)/2 (2.7)/0 (0)
Treatment	
RASB	56 (75.7)
Days between renal biopsy and steroid therapy	12 [1–25]
Oral PSL	61 (82.4)
mPSL therapy	47 (63.5)

Values are presented as the mean (± SD), the median [IQR], and numbers (%)

*HSPN* Henoch- Schönlein purpura nephritis, *eGFR* estimated glomerular filtration rate, *U-RBC* urinary red blood cell sediments, *HPF* high power field, *M* mesangial hypercellularity, *E* endocapillary proliferation, *S* segmental sclerosis/adhesion, *T* tubular atrophy/interstitial fibrosis, *C* cellular or fibrocellular crescents, *JHC* Japanese histologic classification, *ISKDC* International Study of Kidney Disease in Children, *RASB* renin-angiotensin system blockers, *PSL* prednisolone, *mPSL* methylprednisolone

Histologic characteristics are also shown in Table 1. Findings consistent with M1, E1, and S1 were observed in 5 (6.8%), 38 (51.4%), and 38 (51.4%) patients, respectively. In patients with E1, the median number of glomeruli with endocapillary proliferation was 14.3% [IQR: 8.03–22.4]. Findings consistent with T1 and T2 were observed in 14 (18.9%) and 4 (5.4%) patients, respectively. Crescents (ISKDC grade III + IV + V) were identified in 54 (73.0%) patients, including two (3.7%) with crescents in ≥50% of glomeruli (ISKDC grade IV + V). Findings consistent with C1 and C2 were found in 35 (47.3%) and 17 (23.0%) patients, respectively. HG I, II, and III in the JHC were diagnosed in 39 (52.7%), 27 (36.5%), and 8 (10.8%) patients, respectively.

Table 2 presents correlations between the different histologic variables. The strongest correlation was found between the Oxford C lesion and ISKDC classification (*r* = 0.81). Moreover, JHC strongly correlated with Oxford T (*r* = 0.56) and C (*r* = 0.62) and ISKDC classification (*r* = 0.55).

**Table 2:** Spearman correlations between histologic variables in 74 adults with HSPN

	E	S	T	C	JHC	ISKDC
M	-0.28 (*p* = 0.017)	−0.061 (*p* = 0.61)	−0.034 (*p* = 0.78)	−0.28 (*p* = 0.017)	−0.15 (*p* = 0.19)	− 0.27 (*p* = 0.019)
E		0.026 (*p* = 0.82)	− 0.046 (*p* = 0.70)	0.40 (*p* < 0.001)	0.11 (*p* = 0.34)	0.40 (*p* < 0.001)
S			0.12 (*p* = 0.32)	0.17 (*p* = 0.14)	0.21 (*p* = 0.069)	0.23 (*p* = 0.050)
T				0.098 (*p* = 0.41)	0.56 (*p* < 0.001)	0.26 (*p* = 0.027)
C					0.62 (*p* < 0.001)	0.81 (*p* < 0.001)
JHC						0.55 (*P* < 0.001)

*HSPN* Henoch- Schönlein purpura nephritis, *M* mesangial hypercellularity, *E* endocapillary proliferation, *S* segmental sclerosis/adhesion, *T* tubular atrophy/interstitial fibrosis, *C* cellular or fibrocellular crescents, *JHC* Japanese histologic classification, *ISKDC* International Study of Kidney Disease in Children

Table 3 shows a comparison of the clinical features by three pathological classifications. Oxford M is not shown because there were very few M1 lesions. There were significant age-related differences in Oxford T and C lesions, and JHC. eGFR varied significantly by T lesion, JHC, and ISKDC classification. Proteinuria varied significantly by Oxford E and C lesions, and ISKDC classification.

**Table 3:** Comparison of clinical features according to three pathological classifications in 74 adults with HSPN

Factor	Age (years)	*P* values	eGFR (mL/min/1.73m^2^)	*P* values	Proteinuria (g/day)	*P* values
E0	47.4 ± 18.0	0.85	77.1 ± 29.2	0.83	0.77 [0.48–1.32]	< 0.001
E1	48.2 ± 17.0		75.7 ± 22.4		1.83 [1.40–4.58]	
S0	46.9 ± 18.1	0.65	77.2 ± 26.6	0.80	1.38 [0.78–2.44]	0.74
S1	48.7 ± 16.8		75.6 ± 25.4		1.41 [0.64–2.25]	
T0	45.6 ± 17.3	0.048	82.5 ± 23.5	< 0.001	1.41 [0.63–2.32]	0.69
T1 or 2	54.8 ± 16.3		57.3 ± 23.7		1.29 [0.74–2.44]	
C0	44.6 ± 19.6	0.016	84.5 ± 30.1	0.098	0.60 [0.44–1.07]	< 0.001
C1	44.7 ± 15.9		76.1 ± 23.5		1.50 [0.99–2.04]	
C2	58.3 ± 13.7		66.6 ± 21.9		2.30 [1.70–4.89]	
JHC I	42.5 ± 17.7	0.005	85.5 ± 24.8	< 0.001	1.10 [0.57–1.84]	0.10
JHC II or III	53.7 ± 15.2		66.2 ± 23.2		1.70 [0.96–2.55]	
ISKDC I or II	43.8 ± 20.2	0.22	87.7 ± 28.5	0.020	0.67 [0.45–1.12]	0.0052
ISKDC III or IV	49.3 ± 16.2		72.2 ± 23.6		1.70 [0.98–2.49]	

*HSPN* Henoch- Schönlein purpura nephritis, *eGFR* estimated glomerular filtration rate, *E* endocapillary proliferation, *S* segmental sclerosis/adhesion, *T* tubular atrophy/interstitial fibrosis, *C* cellular or fibrocellular crescents, *JHC* Japanese histologic classification, *ISKDC* International Study of Kidney Disease in Children

### Renal outcome study

**Fig. 1:**
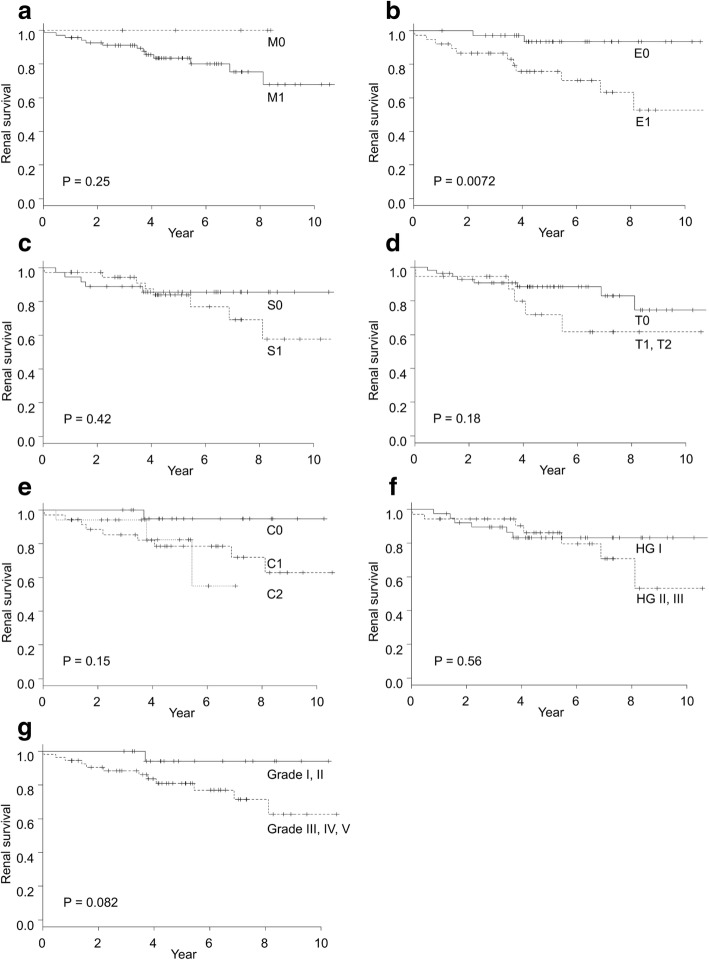
Kaplan–Meier survival curve analyses of event-free renal survival by each pathological classification. **a** Mesangial hypercellularity (M0 vs. M1). **b** Endocapillary proliferation (E0 vs. E1). **c** Segmental sclerosis/adhesion (S0 vs. S1). **d** Tubular atrophy/interstitial fibrosis (T0 vs. T1 + T2). **e** Severity of cellular or fibrocellular crescent formations (C0 vs. C1 vs. C2). **f** JHC (HG I vs. HG II + III). **g** Existence of crescents (ISKDC grades I + II vs. III + IV). Only the Oxford E1 lesion (*p* = 0.0072) was significantly associated with a poor renal survival

During a mean follow-up period of 68.0 ± 33.0 months, fourteen patients (18.9%) reached the renal outcome, defined by a 30% decline in eGFR from the baseline. Among them, only one patient developed ESKD after 11 years following the kidney biopsy. All patients who showed a ≥ 30% decline in eGFR had received oral PSL. Kaplan–Meier survival curves are shown in Fig. 1. The log-rank test revealed significant between-group differences in event-free renal survival in patients with E0 and E1 lesions (*p* = 0.0072). The M, S, T, and C lesions, ISKDC classification, and JHC did not show a significant association with renal outcome. In univariate Cox regression analysis, Oxford E lesion (HR: 6.13, 95% CI: 1.36–27.7) and patient age (HR: 1.64, 95% CI: 1.17–2.30) were significantly associated with renal outcome (Table 4). Male sex and C lesion showed tendencies of association with poor renal outcome, but these tendencies were not statistically significant. Table 4 presents multivariate models adjusted for clinical and pathological factors. In model 1, Oxford E lesion (HR: 8.94, 95% CI: 1.44–55.7) and patient age (HR: 1.58, 95% CI: 1.15–2.18) were significantly associated with an increased risk of poor renal outcome after adjustment for clinical factors (age, sex, and proteinuria). S lesion, T lesion, C lesion, ISKDC classification, and JHC were not associated with an increased risk of poor renal outcome after adjustment for clinical factors. In model 2, Oxford E lesion (HR: 6.71, 95% CI: 1.06–42.7) and patient age (HR: 1.57, 95% CI: 1.12–2.21) were significantly associated with an increased risk of poor renal outcome after adjusting for clinical factors, T lesion, and C lesion.

**Table 4:** Univariate and multivariate Cox regression analyses of factors associated with renal outcome

Variables	Univariate model	*P* value	Multivariate model 1	*P* value	Multivariate model 2	*P* value
	HR (95%CI)		HR (95%CI)		HR (95%CI)	
Age (per 10 years)	1.64 [1.17–2.30]	0.0040	1.58 [1.15–2.18]	0.0052	1.57 [1.12–2.21]	0.0095
Sex (male)	3.04 [0.93–9.91]	0.065	2.56 [0.75–8.73]	0.13	2.92 [0.74–11.6]	0.13
Diabetes	1.21 [0.16–9.44]	0.85				
Hypertension	1.30 [0.42–3.97]	0.65				
eGFR (per 10 mL/min/1.73 m^2^)	0.91 [0.73–1.14]	0.41				
mPSL therapy	1.53 [0.47–4.98]	0.48				
Proteinuria (≥ 1 g/day)	2.16 [0.59–7.88]	0.24	0.49 [0.099–2.43]	0.38	0.52 [0.11–2.42]	0.41
E1 lesion	6.13 [1.36–27.7]	0.018	8.94 [1.44–55.7]	0.019	6.71 [1.06–42.7]	0.044
S1 lesion	1.58 [0.52–4.84]	0.42				
T1 and 2 lesions	2.13 [0.69–6.52]	0.19			2.57 [0.67–9.77]	0.17
C1 lesion (vs. C0 lesion)	5.85 [0.74–46.2]	0.094			2.88 [0.29–29.1]	0.37
C2 lesion (vs. C0 lesion)	6.08 [0.62–59.3]	0.12			2.76 [0.23–33.2]	0.42
JHC II or III (vs. JHC I)	1.39 [0.46–4.14]	0.56				
ISKDC grade III or IV lesion (vs. grade I or II)	5.09 [0.66–39.1]	0.12				

*HR* hazard ratio, *CI* confidence interval, *eGFR* estimated glomerular filtration rate, *mPSL* methylprednisolone, *E* endocapillary proliferation, *S* segmental sclerosis/adhesion, *T* tubular atrophy/interstitial fibrosis, *C* cellular or fibrocellular crescents, *JHC* Japanese histologic classification, *ISKDC* International Study of Kidney Disease in Children

## Discussion

In this study, we retrospectively evaluated renal pathological severity in 74 consecutive patients with HSPN at fourteen different hospitals according to the ISKDC classification, Oxford classification 2016, and JHC. In the Oxford classification 2016, E lesion was significantly associated with an increased risk of poor renal outcome, but the other Oxford lesions including C lesion were not. The JHC and ISKDC classification were not associated with renal outcome in our study. To our knowledge, this is the first report that simultaneously analyzes the Oxford classification 2016, JHC, and the ISKDC classification for their association with renal outcome in adults with HSPN.

One study evaluated adult HSPN pathology according to the Oxford classification 2009. Kim et al. reported that E and T lesions were significantly associated with poor renal outcome in 61 adults with HSPN during a median follow-up period of 49.3 months [13]. Our study also showed that E lesion had an unfavorable influence on renal survival, even in patients with HSPN receiving immunosuppressive therapy. On the other hand, E lesion was associated with poor renal prognosis in IgAN patients with no immunosuppressive therapy [14, 23], but not in those receiving immunosuppressive therapy [14]. These reports indicate that HSPN patients with an E1 lesion are more resistant to immunosuppressive therapy than IgAN patients with an E1 lesion. However, the rates of glomerular lesions with endocapillary proliferation were almost the same in patients with IgAN and those with HSPN. In IgAN patients with an E1 lesion, the median number of glomeruli with endocapillary proliferation was 12% [14]. In our study, in HSPN patients with an E1 lesion, the median number of glomeruli with endocapillary proliferation was 14%. Thus, the characteristics of endocapillary proliferation might have been different between IgAN and HSPN, rather than the number of glomeruli with endocapillary proliferation. Therefore, further studies are needed to determine the mechanism of endocapillary proliferation in HSPN.

In our study, tubular atrophy and interstitial fibrosis were not associated with renal outcome. However, in the HSPN study by Kim et al., T lesion was an independent risk factor for renal outcome. In addition, many studies have shown the utility of Oxford T lesions for predicting the renal outcome in patients with IgAN [14, 19, 24, 25]. We hypothesize that this discrepancy in results was due to aging. HSPN patients with T1 and T2 lesions were significantly older than those with a T0 lesion in our study. Furthermore, the patients we registered were older than those enrolled in the study by Kim et al. (mean age, 47.8 ± 17.4 vs. 34.1 ± 16.4, respectively). Thus, T lesions may not have been related to HSPN severity but may have been associated with arteriosclerosis.

As in a previous study, our study also showed that ISKDC classification was not associated with poor renal outcome [9, 13]. More than 90% of our patients with crescent formation were classified as ISKDC classification grade III (crescents in < 50% of glomeruli) which is consistent with the findings of a previous study [9]. Thus, the ISKDC classification, based on the degree of crescent formation, is not useful in adult HSPN.

We also compared renal outcomes in groups defined by the Oxford C lesion. The C lesion was added to the Oxford classification in 2016 [16]. Haas et al. reported that cellular and fibrocellular crescents (C1 and C2 lesions) were associated with an increased risk of a poor renal outcome in IgAN patients who did not receive immunosuppressive therapy. Furthermore, the presence of these crescents in over 25% of glomeruli (C2 lesion) was associated with a poor renal outcome in all patients with IgAN [21]. However, the C lesion was not significantly associated with poor renal prognosis in our study. We hypothesize that this discrepancy in results occurred because we started immunosuppressive therapy early. In other HSPN studies, the median interval between the onset of purpura and time of renal biopsy was 112 days [13], and the median interval between the kidney biopsy and steroid administration was 9.6 months [26]. In our study, the median interval between the onset of purpura and time of renal biopsy was 53 days, and the median interval between the kidney biopsy and steroid administration was 12 days. As both intervals were much shorter than those in previous studies [13, 26], it is possible that Oxford C lesion was not associated with renal outcome due to this.

We found that JHC was not associated with renal outcome. JHC requires an evaluation of the degree of glomerular histologic changes including cellular, fibrocellular, and fibrous crescents; segmental sclerosis; and global sclerosis [17]. Three facts may explain why JHC was not associated with renal outcome. First, JHC does not include endocapillary proliferation, which was an independent risk factor for poor renal outcome in this study. Second, JHC includes segmental sclerosis and cellular or fibrocellular crescent. In our study, these histological features were not associated with poor renal outcome. Third, JHC was strongly correlated with Oxford T score (*r* = 0.56), which was not an independent predictor for renal outcome in our study. Thus, JHC is unsuitable for the prediction of renal outcome in adults with HSPN.

Our study was limited by its retrospective study design and the limited number of cases. The observed number of renal outcomes in this study yielded a statistical power of approximately 65% for detecting a HR of 3 at the 5% significance level in all patients with HSPN. Although HSPN is more frequently observed in Asian countries than in other countries, adult HSPN is rare among kidney diseases. This is because the incidence of adult HSPN is between one-thirtieth to one-half of the incidence of pediatric HSPN [27], and is about one-tenth of the incidence of adult IgAN [22, 28]. Thus, it was difficult to register a sufficient number of adult patients with HSPN for studies. Therefore, prospective and multinational cohort studies are needed.

## Conclusions

In conclusion, endocapillary proliferation was significantly associated with poor renal outcome in adults with HSPN, even in patients receiving immunosuppressive therapy. Other Oxford classification lesions including C lesions, JHC, and ISKDC classification were not associated with renal outcome.

## Additional file

Additional file 1: Data set of adult HSPN patients (39 females, 35 males, mean age 47.8 ± 17.4 years, and all Japanese). (XLSX 12 kb)

## Abbreviations

BP: Blood pressure
C: Cellular or fibrocellular crescents
CI: Confidence interval
CKD: Chronic kidney disease
E: Endocapillary proliferation
eGFR: Estimated glomerular filtration rate
ESKD: End-stage kidney disease
EULAR/PRINTO/PRES: European League Against Rheumatism/Pediatric Rheumatology International Trials Organization/Pediatric Rheumatology European Society
HG: Histologic grade
HR: Hazard ratio
HSP: Henoch-Schönlein purpura; HSPN: Henoch-Schönlein purpura nephritis
IgAN: IgA nephropathy
IQRs: Interquartile ranges
ISKDC: International study of kidney disease in children
JHC: Japanese Histologic classification
M: Mesangial hypercellularity
S: Segmental sclerosis/adhesion
SD: Standard deviation
T: Tubular atrophy/interstitial fibrosis
